# A multilevel scenario based predictive analytics framework to model the community mental health and built environment nexus

**DOI:** 10.1038/s41598-021-96801-x

**Published:** 2021-09-02

**Authors:** Sayanti Mukherjee, Emmanuel Frimpong Boamah, Prasangsha Ganguly, Nisha Botchwey

**Affiliations:** 1 Department of Industrial and Systems Engineering, School of Engineering and Applied Sciences, University at Buffalo - The State University of New York, Buffalo, NY 14260 USA; 2 Department of Urban and Regional Planning, School of Architecture and Planning, University at Buffalo - The State University of New York, Buffalo, NY 14214 USA; 3 School of City & Regional Planning, College of Design, Georgia Institute of Technology, Atlanta, GA 30332 USA

**Keywords:** Risk factors, Health policy, Public health, Applied mathematics, Computer science, Scientific data, Statistics

## Abstract

The built environment affects mental health outcomes, but this relationship is less studied and understood. This article proposes a novel multi-level scenario-based predictive analytics framework (MSPAF) to explore the complex relationships between community mental health outcomes and the built environment conditions. The MSPAF combines rigorously validated interpretable machine learning algorithms and scenario-based sensitivity analysis to test various hypotheses on how the built environment impacts community mental health outcomes across the largest metropolitan areas in the US. Among other findings, our results suggest that declining socio-economic conditions of the built environment (e.g., poverty, low income, unemployment, decreased access to public health insurance) are significantly associated with increased reported mental health disorders. Similarly, physical conditions of the built environment (e.g., increased housing vacancies and increased travel costs) are significantly associated with increased reported mental health disorders. However, this positive relationship between the physical conditions of the built environment and mental health outcomes does not hold across all the metropolitan areas, suggesting a mixed effect of the built environment’s physical conditions on community mental health. We conclude by highlighting future opportunities of incorporating other variables and datasets into the MSPAF framework to test additional hypotheses on how the built environment impacts community mental health.

## Introduction

The physical and socio-economic aspects of the built environment impact population mental health outcomes of a community. The physical aspects include human-created infrastructure systems, such as transportation and housing infrastructure systems, which support the functioning of people within a community^1^. The socio-economic aspects refer to the economic, racial and ethnic, and relational conditions that may influence a person’s ability to function well, both physically and psychologically, within their communities. Studies have examined how such physical and socio-economic aspects of the built environment impact a community’s overall health and well-being in terms of crime rates^2^, educational performance, property values^3^, and various health outcomes such as obesity, heart disease, cancer, stroke, respiratory disease, diabetes, and suicide rates^4–7^. More specifically, understanding and predicting health outcomes as a function of the built environment is a significant focus among urban planning, public health and allied professionals. The SARS-COV-2 pandemic has further exacerbated the urgency to understand how such aspects of the built environment influence health outcomes. Examples include—studying the reasons for faster disease spread within the vulnerable and minority population based on the socio-economic conditions and physical setting of their surrounding built environment^8,9^, investigating how the different conditions within the built environment (e.g., sanitation conditions and closed and open areas) or different types of physical surfaces (e.g., metal and other solid surfaces, and water) aid in the spread of the virus^10,11^.

Mental health is one of the specific health outcomes impacted by the built environment. Mental illness or disorder contribute significantly to the global burden of disease, accounting for 32.4% of years lived with disability (YLDs) and 13.0% of disability-adjusted life-years (DALYs), globally^12^. As of 2016, global estimates revealed that mental discourses (e.g., chronic depression, anxiety, substance use disorders) were significant contributors to disability in young adults; depressive and anxiety disorders were high among females, while substance use and autism spectrum disorders were high among males^13^. In the US, suicide ideation in adults is increasing, with 10.3 million adults diagnosed with severe thoughts of suicide^14^, and over two million youth having severe depression^15^. In fact, depression and hopelessness are the key predictors of suicide ideation and attempts in young adults^6^. Based on the commissioning report on mental health and the sustainable development goals (SDGs)^16^, mental health is considered a “global public good”, but both developed and developing countries struggle to understand and address the complex physical, social, and environmental influences that interact with genetic, neuro-developmental, and psychological processes driving the mental health and well-being of people^16^.

Although the built environment impacts mental health, there are gaps in the literature about the complex and non-linear relationships between mental health outcomes and the built environment. Studies examining the socio-economic determinants of mental health have shown that poverty, childhood adversity, and violence are the key risk factors of mental health disorders^17^. Studies have also indicated a disparity among large and medium/small metropolitan areas’ suicide rates with the latter being higher than the former^7^. Variations in population demographics and socio-economic factors such as unemployment rates, household income, and climate are the key factors associated with such disparities^7^. Other studies have also looked at the link between mental health and low quality of care for mental health disorders as well as human rights abuses^18^. However, there are gaps in the literature about the link between the various aspects of built environment and mental health outcomes. In what they delineate as the “neighborhood domain”, the commissioning report on the SDGs and mental health indicates that poorly planned or deteriorating neighborhoods (e.g., housing vacancy and declining quality of housing and community infrastructure) pose mental health challenges on individual-level biological markers^16^.

Various studies attempt to explain the link between population mental health and the built environment. For example, one study found that adolescents living in physically deteriorated neighborhoods had more health problems, including depression and anxiety than those living in ordered neighborhoods^19^. Another research project that studied 1355 residents in the New York City found that populations living in poor quality neighborhoods had a greater likelihood of experiencing chronic depression, after controlling for their income, race/ethnicity, age, and neighborhood-level income^20^. A cross-sectional study of adults (16 years and above) residing in north London showed that the prevalence of depression had a statistically significant relationship with living in areas characterized by deck access homes (i.e., abundant with graffiti and without shared recreational spaces), after adjusting for individuals’ internal characteristics of their dwellings and socio-economic status^21^. A systematic review of 45 studies reveals that 37 reported at least one built environment characteristic associated with depression or depressive symptoms^22^.

However, despite some advancements in understanding the interplay between the built environment and mental health, there are limited methodological frameworks to parse the nonlinear relationships between the built environment and mental health outcomes^23^, which this article aims to contribute. For instance, apart from genetic, lifestyle, and physio-psychological factors, mental health is influenced by a complex interplay of the physical and socio-economic aspects of the built environment (e.g., neighborhood decline, transportation conditions, unemployment, income, race, age, social capital, education). The complex, non-linear relationship between the built environment factors and mental health outcomes constrains the traditionally-used linear and static models’ explanatory and predictive abilities^24^. Even though these complex interactions are acknowledged, studies are yet to leverage recent advances in big data analytics to explore such complexities.

In this study, we demonstrate a novel data-driven approach to study the complex associations between mental health of adults within a metropolitan community and the physical and socio-economic aspects of the built environment, thus guiding how properly planned neighborhoods may improve the overall mental health outcomes of the adult population within a community. Specifically, we develop and employ a novel methodological framework, a multi-level scenario-based predictive analytics framework (MSPAF), to explore the complex relationships between mental health outcomes and conditions in the built environment. The MSPAF combines rigorously validated interpretable machine learning algorithms and scenario-based sensitivity analysis to test several hypotheses on how the built environment affects mental health outcomes across the largest metropolitan areas in the US. The scenario-based analysis predicts how the community mental health outcomes in these metropolitan areas change under plausible perturbations of various built environment factors.

## Results

### Predictive performance of interpretable machine learning models and model selection

This study leveraged a library of supervised interpretable machine learning models to assess the associations between community mental health outcomes and, the built environment’s physical and socio-economic aspects. Interpretable machine learning models, ranging from parametric, semi-parametric to non-parametric models, vary widely in their degree of complexity, robustness, flexibility, and interpretability (discussed further in “Overview of statistical learning” section)^25,26^. The statistical learning techniques are used in different research areas, such as energy demand modeling^27^, infrastructure vulnerability assessment^28^ or crime prediction^2^. The parametric modeling technique (e.g., linear regression models), where a parametric function is fitted to the training data (e.g., via mechanisms such as least-squares), is the most popular modeling approach in healthcare research. Although such models are simple and easier to interpret, they often fail to approximate the true function since real relationships are often not linear. On the other hand, non-parametric data-driven models do not make any unrealistic assumptions about the functional form, thereby better approximating the true functional form. However, flexibility comes at the cost of interpretability^25^. Although such data-driven non-parametric algorithms have seen their wide application in various domains of risk assessment such as crime risk modeling^2^, energy supply inadequacy risk^27,29–34^, infrastructure risk assessment^35^, natural disaster risk assessment^28,36,37^, among others, these methods are significantly under-explored in healthcare research despite their robustness and flexibility. To bridge this gap, this study assessed the predictive performance of eight interpretable machine learning models ranging from parametric to non-parametric—generalized linear model (GLM)^38^, ridge regression (RR)^39^, lasso regression (LR)^40^, generalized additive model (GAM)^41^, multivariate adaptive regression splines (MARS)^42^, gradient boosting method^43^, random forest (RF)^44^, and Bayesian additive regression tree (BART)^45^. Leveraging an 80–20 randomized percentage holdout cross-validation technique, we estimated the generalization performances of the models and selected the model that outperformed all the other models in terms of both in-sample goodness-of-fit and out-of-sample predictive accuracy (see “Overview of statistical learning” section). The model performances of the various models are depicted in the Table 1. Our results indicate that BART outperformed all the other models which is, thus, leveraged for the relevant statistical inferencing (see “Key factors attributing to socio-economic and physical aspects of the built environment” section).

**Table 1:** Model performance comparison.

Model	$R^2$	In-sample		Out-of-sample	
		RMSE	MAE	RMSE	MAE
Generalized linear model	0.987	0.387	0.295	0.389	0.295
Ridge regression	0.971	0.584	0.457	0.588	0.459
Lasso regression	0.962	0.661	0.517	0.665	0.519
Generalized additive model	0.991	0.315	0.238	0.319	0.240
Multivariate adaptive regression splines [MARS]	0.983	0.441	0.328	0.442	0.328
MARS [degree=2]	0.982	0.454	0.343	0.456	0.344
MARS [degree=3]	0.982	0.454	0.342	0.456	0.343
MARS [degree=3; penalty=2]	0.981	0.454	0.361	0.456	0.343
Random forest	0.996	0.199	0.139	0.493	0.347
Gradient boosting method	0.994	0.261	0.197	0.309	0.282
Bayesian additive regression trees	0.997	0.182	0.136	0.221	0.159
Null	NA	3.382	2.773	3.386	2.774

### Key factors attributing to socio-economic and physical aspects of the built environment

We leveraged the variable importance plot (VIP) (see Supplementary Information) and the partial dependence plots (PDPs) to identify the key built environment predictors of mental health outcomes, and evaluated their associated relationships (see “Overview of statistical learning” section for mathematical details of the VIP and PDP). For our analysis, we also controlled for behavioral and underlying health conditions (e.g., smoking habit, principal components of underlying physical health conditions) that significantly influence mental health outcomes. Since this study focuses on the built environment factors, our subsequent discussions will focus on the built environment’s physical and socio-economic aspects, which remain under-explored and are central to this article.

**Figure 1:**
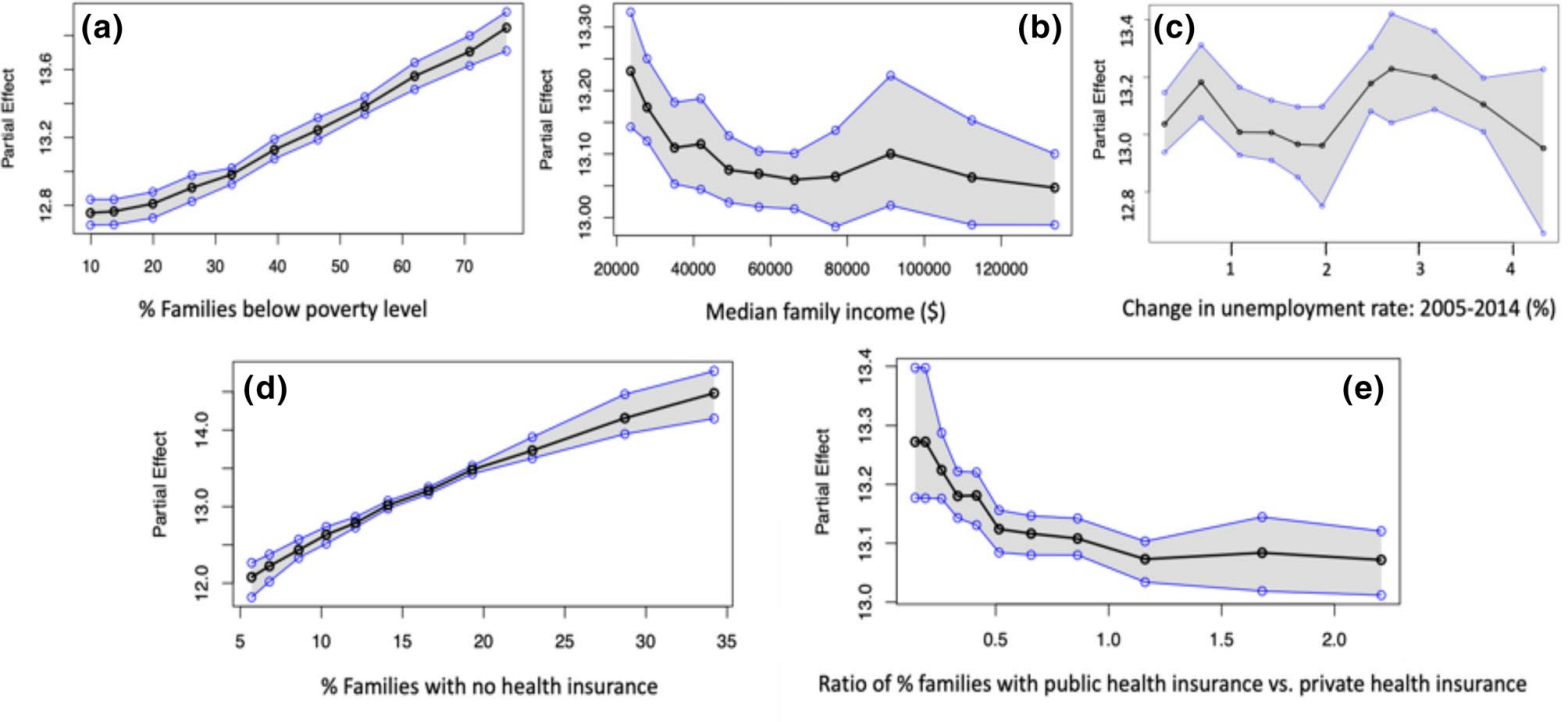
Partial Dependence Plots (PDPs) of the key socio-economic aspects of the built environment: (**a**) $\%$ of families below poverty level, (**b**) median family income, (**c**) $\%$ change in unemployment rate, (**d**) $\%$ families with no health insurance, (**e**) ratio of $\%$ of families with public health insurance vs. private health insurance. In each sub-figure, on the $x{\text{-}}axis$, the values of the particular independent variable is plotted, and on the $y{\text{-}}axis$, the partial effect of the independent variable on the response variable is depicted. The black curve is the average partial effect of the predictor variable and blue lines indicate the $95\%$ confidence intervals.

Partial dependence plots (PDPs) of the socio-economic aspects of the built environment, are depicted in Fig. 1. The PDP of poverty, shown in Fig. 1a, indicates a strong positive correlation with mental health outcomes. This relationship suggests that as the percentage of families below the poverty level increases from 10 to 80%, the percentage of adults ($$>18$$ years) reporting poor mental health (mental health not good for $$\ge 14$$ days) increases from 12.8 to 13.8% in the community on average. The narrow confidence interval (represented by the shaded grey area) indicates that the estimates are associated with less uncertainty. Other significant factors in this category include economic variables such as median family income and change in the unemployment rate (2005–2014). The partial dependence plot of median family income (Fig. 1b) shows a negative correlation. More specifically, we observe that as the median family income decreases from around $130,000 to $20,000, the percentage of adults reporting mental health disorders increases from 13.0 to 13.3% on average in a community. However, the wider confidence interval around the larger income values indicates that the estimated mental health outcomes for adults in the higher income range significantly vary. On the other hand, the relationship between unemployment changes and the percentage of adults reporting poor mental health is relatively uncertain Fig. 1c. Besides the economic status of a community, access to medical insurance plays a major role in predicting the community mental health outcomes. The PDP of the percentage of families with no health insurance (Fig. 1d) shows that it has a strong positive correlation with mental health outcomes. It is observed that as the percentage of families with no health insurance in a community increases from 5 to 35%, the percentage of adults reporting poor mental health increases from 12 to 14.5%. The narrow confidence interval indicates lower uncertainty and variations in the estimated relationship across the US metropolitans. Our results also suggest that the insurance type plays a major role in influencing mental health of adults within a metropolitan community. The PDP of insurance type, representing the ratio of percentage of families with public health insurance to private health insurance, is plotted in Fig. 1e. From the plot, we observe that as the proportion of families having public health insurance compared to that having private health insurance approximately doubles, the percentage of adults reporting poor mental health declines from 13.4 to 13.0% on average. The decreasing trend indicates that increased access to public health insurance is associated with decreased mental health disorders reported by adults in a metropolitan community on average.

**Figure 2:**
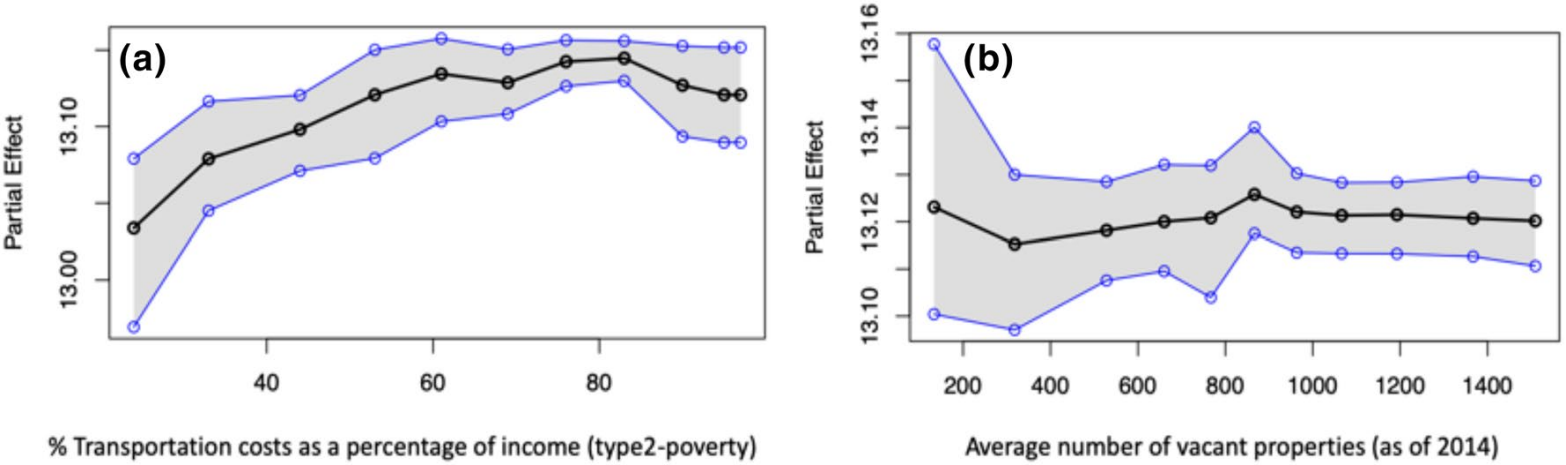
Partial Dependence Plots (PDPs) of the key physical aspects of the built environment: (**a**) $\%$ of the transportation cost as a percentage of income and (**b**) average number of vacant properties. In each sub-figure, on the $x{\text{-}}axis$, the values of the particular independent variable is plotted, and on the $y{\text{-}}axis$, the partial effect of the independent variable on the response variable is depicted. The black curve is the average partial effect of the predictor variable and blue lines indicate the $95\%$ confidence intervals.

Our result shows that transportation or commuting cost (percentage of household income spent on transportation) and the average number of vacant properties, which constitute the built environment’s physical aspect, are the key predictors of community mental health. The PDP of transportation cost shows a positive correlation with poor mental health (Fig. 2a). More specifically, we observe that as household transportation expenditures increase from 20 to 100% on average, the percentage of adults reporting poor mental health increases from 13.0 to 13.10% on average. Although this increment seems small, it should be noted that these numbers only indicate the national average of large metropolitan communities, with some US states experiencing much higher negative impacts than others. Our scenario based sensitivity analysis (refer to “Data and methods” section) emphasizes such variations across the various metropolitan areas in the US states. However, the relationship between average number of vacant properties and community mental health is uncertain (Fig. 2b). We observe a slightly increasing trend in the percentage of adults reporting poor mental health as the average number of vacant properties increases. However, as the trend reaches the threshold point around 900 vacant properties in a community on average, the association flattens, that is, the number of reported mental health issues becomes insensitive to changes in vacant properties at a certain threshold level.

### Projected community mental health burden under plausible perturbations

Having identified the key built environment factors associated with mental health outcomes, we employed a scenario-based sensitivity analysis to understand how mental health outcomes may change under different built environment scenarios. Plausible future scenarios are captured through perturbations of the socio-economic and physical aspects of the built environment. Traditionally, in modern epidemiological studies, the sensitivity and uncertainty analyses for any disease burden and risk factor estimates are conducted using different weighting mechanisms and discount rate techniques^46^. However, due to large degrees of uncertainties associated with value judgments and built environment conditions, the choice of discount rates is challenging and often cannot capture the wide range of future uncertainties^47^. To overcome these challenges, we limited our analysis to statistical perturbation. The statistical perturbation consists of three significant steps described as follows: (1) we statistically perturbed the socio-economic and physical aspects of the built environment, which may lead to increase (e.g., economic growth) or decrease (e.g., economic recession) in the independent variable under consideration; (2) following a general intuition, we hypothesized whether the increase (decrease) in the independent variable leads to better (worse) mental health outcomes and vice-versa; and, (3) leveraging our predictive model, we verify if our hypothesis is valid nationally or only for certain US states (see “Data and methods” section for details on creating scenarios and list of hypotheses summarized in Table 2).

**Table 2:** Summary of the hypotheses.

Independent variable category	Perturbation scenario and implications	Hypothesis index	Hypothesis statement
Economic characteristics	*Economic degradation * increase in % families below poverty level and unemployment rate by $1\sigma$; decrease in median household income by $1\sigma$	*H*1	Economic degradation leads to degradation in community mental health or increase in *K*
	*Economic improvement* decrease in % families below poverty level and unemployment rate by $1\sigma$; increase in median household income by $1\sigma$	*H*2	Economic improvement leads to improvement in community mental health or decrease in *K*
Unavailability of health insurance	*Less unavailability of health insurance* % families with no health insurance decreases by $1\sigma$	*H*3	Less unavailability of health insurance leads to improvement in community mental health or decrease in *K*
	*More unavailability of health insurance* % families with no health insurance increases by $1\sigma$	*H*4	More unavailability of health insurance leads to degradation in community mental health or increase in *K*
Category of health insurance	*Decreased access to public health insurance* Ratio of % families with public vs. private insurance decreases by $1\sigma$	*H*5	Decreased access to public insurance leads to degradation in community mental health or increase in *K*
	*Increased access to public health insurance* Ratio of % families with public vs. private insurance increases by $1\sigma$	*H*6	Increased access to public insurance leads to improvement in community mental health or decrease in *K*
Transportation cost	*Cheaper mode of travel and/or shorter commuting distance to work * annual transportation cost decreases by $1\sigma$	*H*7	Decreased transportation cost leads to improvement in community mental health or decrease in *K*
	*Expensive mode of travel and/or longer commuting distance to work* annual transportation cost increases by $1\sigma$	*H*8	Increased transportation cost leads to degradation in community mental health or increase in *K*
Average number of vacant properties	*People moving into the metropolitan areas (community expanding)* average number of vacant properties decreases by $1\sigma$	*H*9	Decrease in vacancy would lead to improvement in community mental health or decrease in *K*
	*People moving out of the metropolitan areas (community shrinking)* average number of vacant properties increases by $1\sigma$	*H*10	Increase in vacancy would lead to degradation in community mental health or increase in *K*

For illustration purpose, consider *K* represents the community mental health (response variable in our analysis), measured in terms of “% adults aged $> 18$ suffering from poor mental health for $> 14$ days”. Hence, *improvement in mental health* is depicted by a *decrease in* *K*, and *deterioration of community mental health* is observed when there is an *increase in* *K*. The predictor or independent variables under consideration for scenario-based sensitivity analysis are grouped into five categories, viz., (i) economic factors consisting of median household income, $\%$ of population below the poverty level and unemployment rate (ii) percentage of families with no health insurance, (iii) proportions of families having public insurance compared to private insurance, (iv) percentage of transportation cost spent as a $\%$ of household income, and (v) the average number of vacant properties (as of 2014). The first three categories of independent variables capture the socio-economic characteristics of the built environment and the last two categories represent the physical aspects of built environment. We assume, two hypothetical scenarios at a given time in our study: (1) the mean of the distributions of the socio-economic parameters (i.e., economic conditions, access to health insurance, and type of health insurance) and the physical aspects (i.e., travel cost and housing vacancy) of built environment of a community shifts by $+ 1$ standard deviation from their historical mean, which represents the base case or *as-is* scenario; and, (2) the mean of those distributions shift by $- 1$ standard deviation from their historical mean. Note that, these statistical perturbations help to provide important insights regarding the trends of community mental health outcomes under plausible scenarios. However, it should be noted that our framework is generalized enough that it can be used to predict how the community mental health outcomes may change in the future, given the forecasted data on socio-economic aspects and built environment is available. Our study presents the framework illustrating how future mental health outcomes might be affected under various future scenarios. Furthermore, to understand whether such shifts results in a favorable outcome (improved mental health) or not, we compared the projections with the base case scenario of mental health outcomes by constructing ten hypotheses (see Table 2 in “Data and methods” section). Finally, we validated our hypotheses based on our model results and outcomes.

#### The socio-economic aspects of built environment

Overall, our scenario-based sensitivity analysis indicates that the metropolitan areas in the eastern part of the US have poor mental health outcomes. We discuss the observed variations in the community mental health outcomes across the 50 states in the US under the six different scenarios of the socio-economic aspects of built environment—worst- and best-case scenarios of (a) economic condition, (b) lack of health insurance, and (c) access to public health insurance.

##### Economic condition

**Figure 3:**
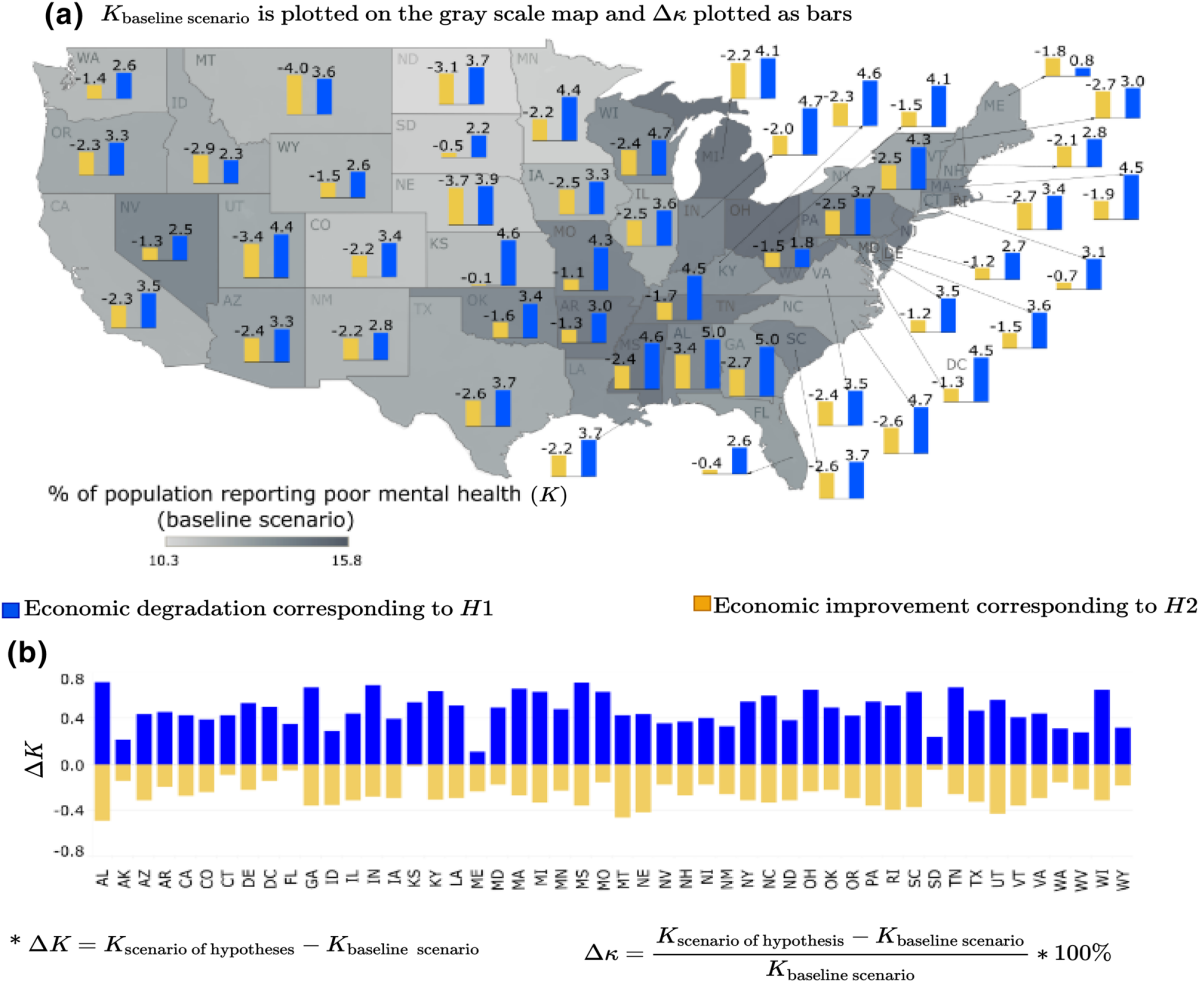
Economic condition scenario for % of adults aged $>18$ years reporting poor mental health for $>14$ days (*K*): (**a**) $\Delta \kappa$ is plotted as the bars and *K* for base line scenario is plotted as gray scale intensity on the US map; and for (**b**) $\Delta K$ is plotted. The maps are created using Tableau 2021.2 (Tableau 2021.2 New Features) and Adobe Illustrator (Software and services for creative business teams | Adobe Creative Cloud for teams).

The economic conditions capture the interplay of poverty, median household income, and unemployment rate of the population in a metropolitan area. Since economic condition comprises three variables, for simplicity the hypothetical scenario is constructed by perturbing all the three variables simultaneously. It was hypothesized that during declining economic conditions, the expected percentage of people reporting poor mental health (*K*) would increase (hypothesis: *H*1), and the opposite effect would be observed during an increase in economic growth/boom (hypothesis: *H*2). The scenario-based analysis conducted herein supports these two hypotheses throughout all the states in the US. As depicted in Fig. 3, when economic depression sets in (blue bars), all the states observe a deterioration in community mental health depicted by $\Delta K >0$. On the other hand, when the community experiences an economic boom (yellow bars), improvement in community mental health is observed depicted by $\Delta K < 0$. The scenario analysis, depicted in Fig. 3, shows that the percentage change (increase or decrease) in reported mental disorders among adults is more pronounced in metropolitan areas within states such as Alabama, Georgia, Indiana, Massachusetts, Kentucky, Michigan, Mississippi, Montana, Ohio, South Carolina, Tennessee, Utah, and Wisconsin. A recent systematic review identifies economic conditions as one of the social determinants of mental health^48^. These conditions are linked to poverty^16,48^, income^49^, and unemployment^50^. The scenario-based analysis confirms some of these earlier studies, but it also goes a step further to provide a metropolitan-level analysis of how improving or declining economic conditions affect the mental health of adults in specific metropolitan areas in the US. Moreover, we also observe that community mental health is more sensitive to economic depression (longer blue bars for economic degradation) than economic boom (shorter yellow bars representing economic growth).

##### Unavailability of health insurance

**Figure 4:**
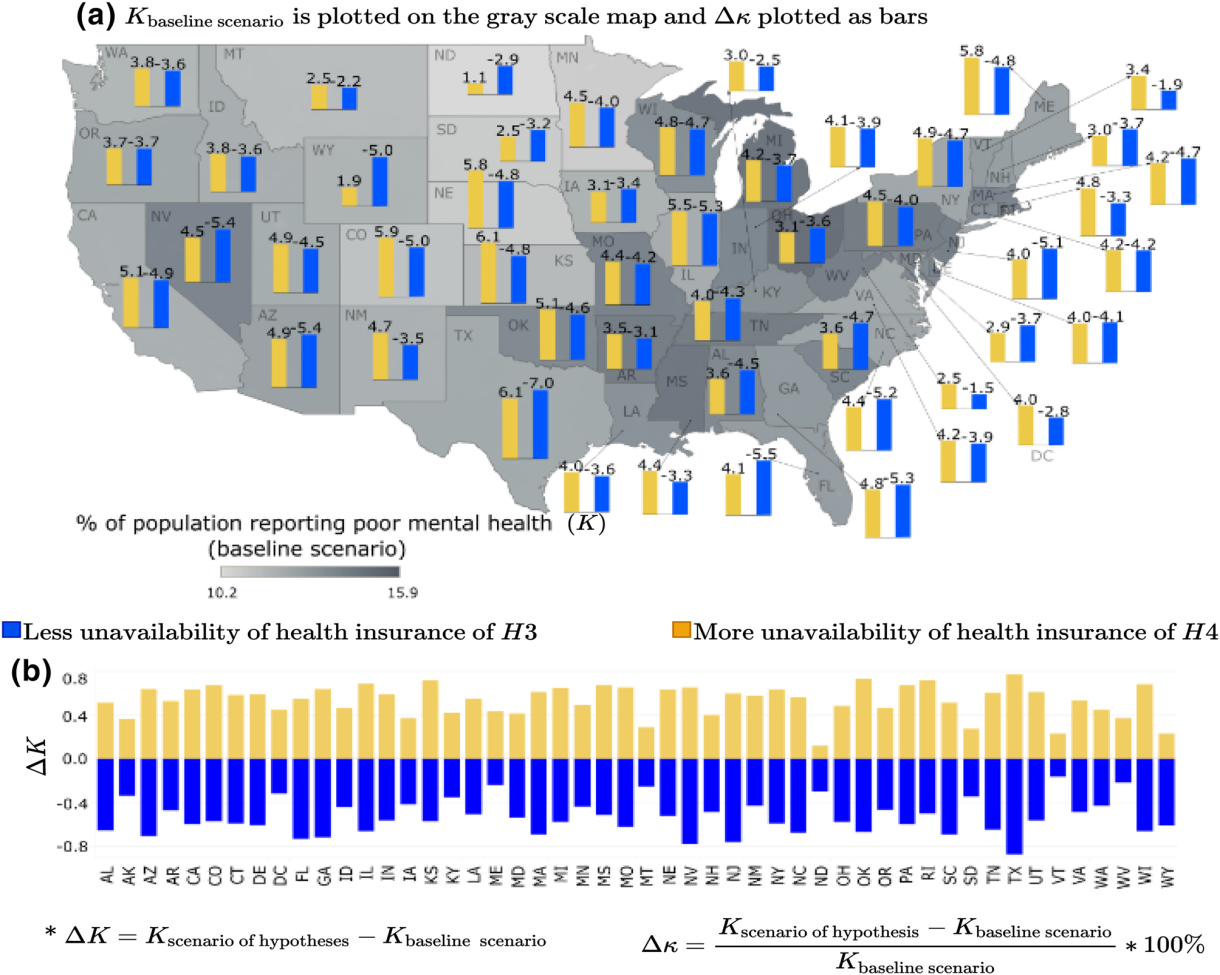
Unavailability of health insurance scenario for % of adults aged $>18$ years reporting poor mental health for $>14$ days (*K*): (**a**) $\Delta \kappa$ is plotted as the bars and *K* for base line scenario is plotted as gray scale intensity on the US map; and for (**b**) $\Delta K$ is plotted. The maps are created using Tableau 2021.2 (Tableau 2021.2 New Features) and Adobe Illustrator (Software and services for creative business teams | Adobe Creative Cloud for teams).

In this case, the variable under consideration is the percentage of families with no health insurance or lack of access (unavailability) to health insurance. It was hypothesized that an overall improvement in community mental health would be observed when the unavailability of mental health will decrease, i.e., more families will have health insurance (hypothesis: *H*3). An opposite effect is expected with increased unavailability of health insurance, or in other words when, more families are being deprived of health insurance, which may lead to worsening mental health problems (hypothesis: *H*4). The scenario-based analysis in Fig. 4 suggests that these two hypotheses generally hold true for all the metropolitan areas across the US, considered in this study. From Fig. 4, we observe that when the percentage of families with no health insurance increases (yellow bars), the number of adults reporting poor mental health in the community (*K*) increases, compared to the baseline scenario. An opposite effect, i.e., decrease in the number of people reporting poor mental health is observed for the scenario depicting higher percentage of families in a community having health insurance (blue bars). However, a change (increase or decrease) in access to health insurance results in a minimum shift in the percentage of adults reporting mental disorders in the metropolitan areas of the states such as Montana, North Dakota, South Dakota, and Vermont. Studies show that states providing better access to mental health insurance minimize suicide rates^51^, but another study found that Australia’s mental health insurance under its “Better Access scheme” has had no significant effect on the mental health of Australians^52^. The underlying logic follows that increasing access to health insurance and, specifically, mental health insurance will likely increase the likelihood of more number of people accessing mental healthcare^53,54^, which will ultimately improve overall mental health outcomes. The scenario-based analysis results contribute to this debate by explicitly looking at how the lack of access to health insurance in general, not only mental health insurance, may contribute to adults’ increasing stress and poor mental health outcomes.

##### Access to public health insurance

**Figure 5:**
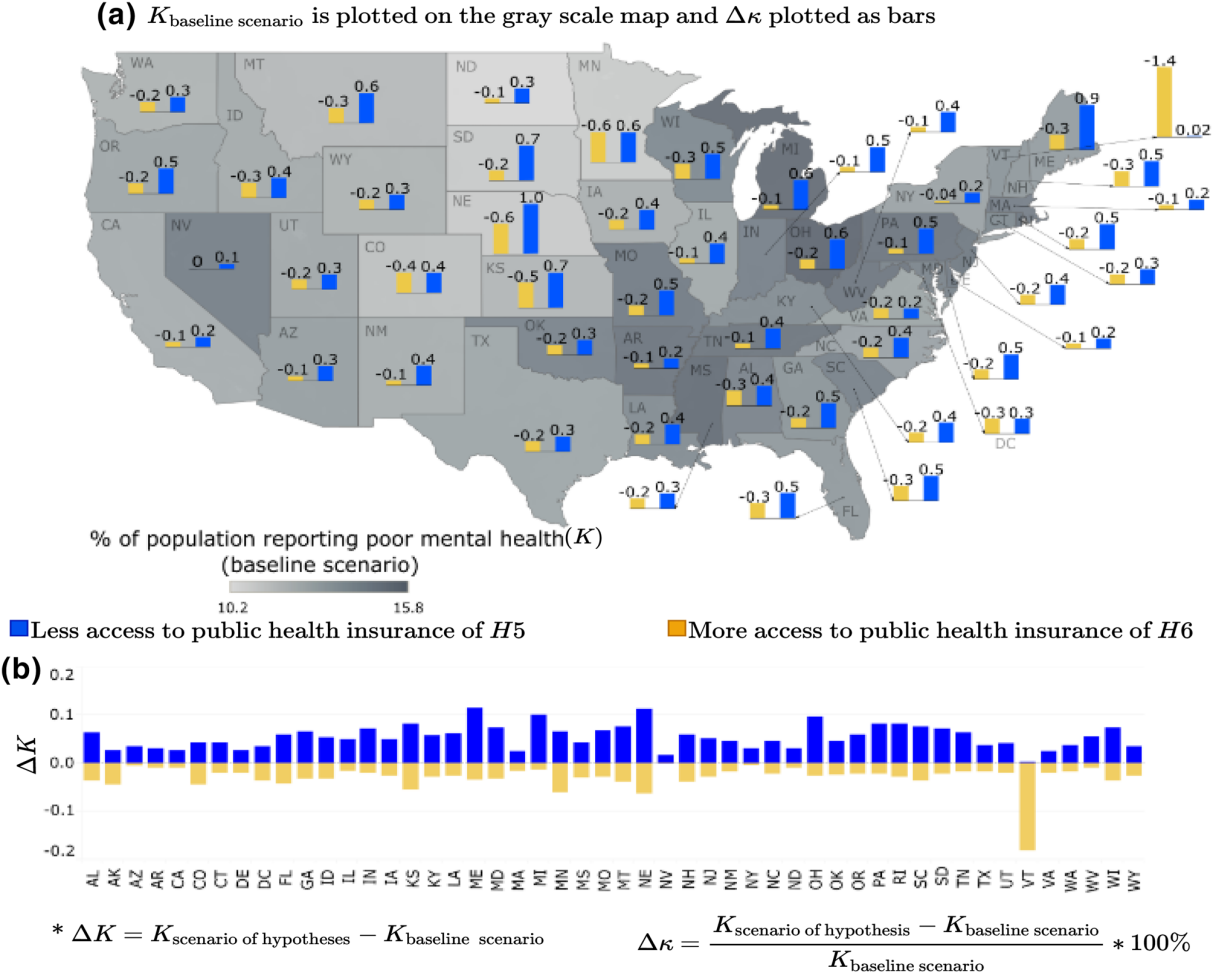
Access to public health insurance scenarios for % of adults aged $>18$ years reporting poor mental health for $>14$ days (*K*): (**a**) $\Delta \kappa$ is plotted as the bars and *K* for base line scenario is plotted as gray scale intensity on the US map; and for (**b**) $\Delta K$ is plotted. The maps are created using Tableau 2021.2 (Tableau 2021.2 New Features) and Adobe Illustrator (Software and services for creative business teams | Adobe Creative Cloud for teams).

Building on the health insurance scenario analysis, we hypothesized prevalence of differential impacts of the two different types of health insurance, i.e., public vs. private health insurance on the community mental health outcomes. Specifically, we hypothesized that with decreased access to public health insurance (i.e., a lower proportion of people with access to public health insurance), the overall mental health of the community would worsen, leading to an increase in *K* (hypothesis: *H*5). The opposite effect of improving mental health would be observed with increased access to public health insurance (hypothesis: *H*6). However, these hypotheses were minimally supported in the scenario results across the states, as depicted in Fig. 5. Although the trend of increasing or decreasing mental health outcomes was found to be consistent across all the states having $\Delta K < 0$ for all the states when access to public health increases (yellow bars) and $\Delta K > 0$ for all the states when access to public health decreases (blue bars), the magnitude of such deviations significantly varies, ranging between $- 0.5\%$ to $+ 1.0\%$. This varying range indicates that the overall mental health outcomes across the US’s metropolitan areas are not very sensitive to the type of health insurance. However, the hypothesis of decreasing *K* with increasing access to public health insurance (hypothesis: *H*6) was significantly supported for the metropolitan areas in Vermont. For context, Vermont was the first state in the US to adopt legislation for universal health care for its residents in 2011, making health insurance and healthcare publicly available to many residents, including free preventative services such as mental health and substance-based disorder services^55^.

#### The physical aspect of the built environment

##### Travel/commuting cost

The scenario-based sensitivity analysis for travel cost—measured by the “% of transportation cost spent as a % of household income”—illustrates the extent to which the commuting cost within sprawling metropolitan areas can impact community mental health outcomes. The hypotheses explored here are—(1) the percentage of adults reporting mental disorders (*K*) would decrease with decreasing travel cost (hypothesis: *H*7) and, (2) the percentage of adults reporting mental disorders (*K*) would increase with increasing travel cost (hypothesis: *H*8). However, our analysis shows that these two hypotheses do not hold for some metropolitan areas in some of the states. For instance, the hypothesis (*H*8) that increased mental health disorders (*K*) are reported as the travel cost increases do not hold in metropolitan areas within the states such as Colorado, Delaware, Maine, Minnesota, Nebraska, North Dakota, Utah, Vermont, and Washington. There is a decrease in mental health disorders reported by adults in these metropolitan areas as the travel costs increase. For metropolitan areas in Washington DC, Maryland, and New Hampshire, an increase or decrease in travel costs has the same effect, i.e., an increase in the percentage of adults reporting mental health disorders. The decrease in mental health disorders as travel cost increases is generally consistent with findings in the literature. An increase in travel cost is often associated with sprawling areas, i.e., travel cost increases with sprawl^56,57^. Some studies found that increasing sprawl (or commuting cost) either had no association with mental health disorders^58,59^ or was positively associated with better mental health, by allowing those living in low-density sprawl areas to enjoy proximity to nature^60,61^. Some studies also found that shorter distances (decreased travel cost) to the city center positively influence subjective wellbeing^62,63^. On the other hand, some studies found that increase sprawl or travel cost negatively impacts mental health, especially for residents living in auto-dependent sprawling neighborhoods with no access to personal vehicles^64^. The mixed results of how travel costs impact mental health outcomes in our analysis resonates with the existing literature, and it signals the need for an in-depth and granular inquiry into how the built environment’s physical aspect impacts mental health outcomes in cities.

**Figure 6:**
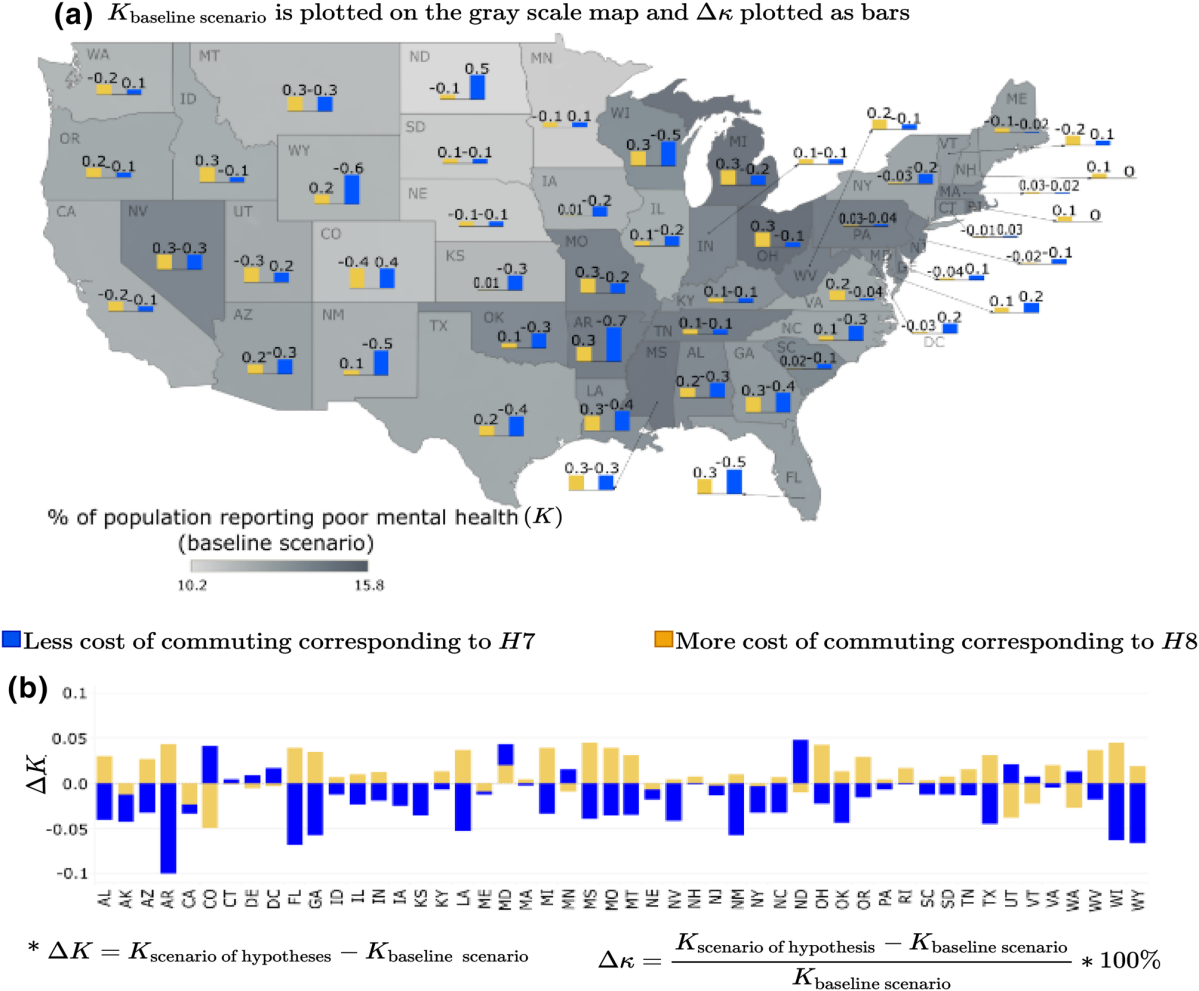
Travel cost scenarios for % of adults aged $>18$ years reporting poor mental health for $>14$ days (*K*): (**a**) $\Delta \kappa$ is plotted as the bars and *K* for base line scenario is plotted as gray scale intensity on the US map; and for (**b**) $\Delta K$ is plotted. The maps are created using Tableau 2021.2 (Tableau 2021.2 New Features) and Adobe Illustrator (Software and services for creative business teams | Adobe Creative Cloud for teams).

##### Housing vacancy

**Figure 7:**
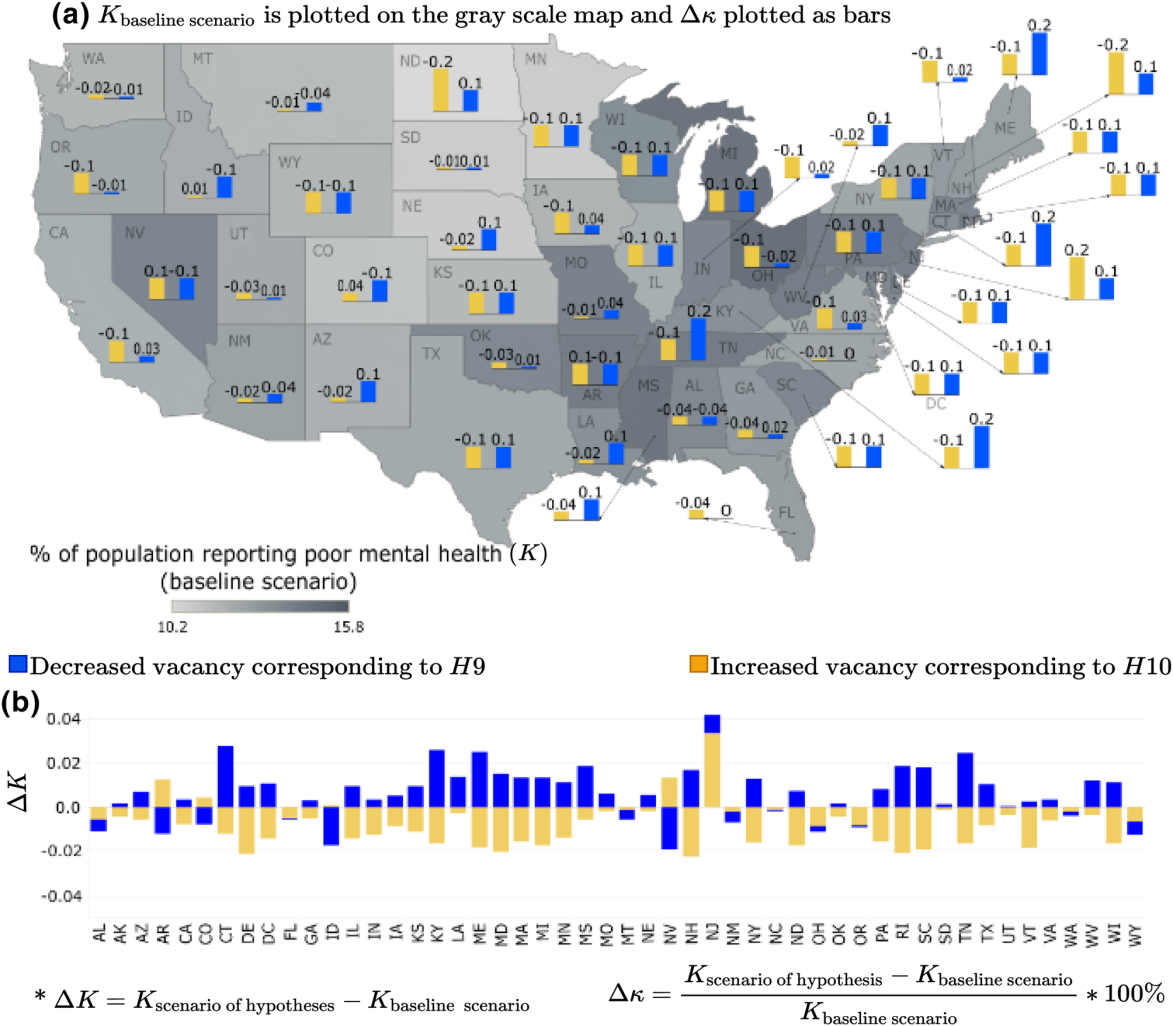
Housing vacancy scenario for % of adults aged $>18$ years reporting poor mental health for $>14$ days (*K*): (**a**) $\Delta \kappa$ is plotted as the bars and *K* for base line scenario is plotted as gray scale intensity on the US map; and for (**b**) $\Delta K$ is plotted. The maps are created using Tableau 2021.2 (Tableau 2021.2 New Features) and Adobe Illustrator (Software and services for creative business teams | Adobe Creative Cloud for teams).

In the housing vacancy scenario, the hypotheses explored in this study investigated the extent to which neighborhood decline impacts mental health outcomes in the metropolitan areas across the 50 states in the US. Specifically, we hypothesized that a decrease in housing vacancy would lead to a decline in adults reporting poor mental health in metropolitan areas or *K* (hypothesis: *H*9). On the other hand, an increase in vacant properties or a decline in neighborhood size was expected to increase the percentage of adults reporting mental disorders (hypothesis: *H*10). However, overall, these hypotheses were not supported in our study. As depicted in Fig. 7, when the housing vacancy increased (yellow bars), most metropolitan areas across the US states experience an improvement or deterioration in the community mental health (*K*). On the other hand, when a community is expanding, attributed by decreased vacancy (blue bars), most of the metropolitan areas see an increase in the percentage of adults reporting poor mental health (*K*). This result may be an outcome of the “Behavioral Sink” phenomenon^65,66^. However, for some states the reverse phenomenon has been observed. For instance, when the vacancy is decreasing, metropolitan areas of some states such as Alabama, Florida, Montana, New Mexico, North Carolina, Ohio, Oregon, Washington, and Wyoming see an improvement in mental health depicted by $\Delta K <0$. On the other hand, when the vacancy is increasing, some states’ metropolitan areas (Arizona, Colorado, Nevada, and New Jersey) see a deterioration of mental health with $\Delta K > 0$. The mixed results from this scenario analysis support our earlier observations related to the transportation cost, emphasizing that there is more to the story when parsing the impacts of the built environment on community mental health outcomes. More granular-level analysis complemented by macro-level analyses might better help unpack how the built environment’s physical conditions at the household, neighborhood, city, and county levels may impact an individual’s mental health.

As discussed, the results depicting community mental health (*K*) sensitivity to housing vacancy are highly varied across the US states. Hence, it is difficult to classify whether a particular scenario of housing vacancy perturbation leads to the *best case scenario*, representing improvement in community mental health unanimously across the US states; or if the perturbation leads to the *worst-case scenario*, where the community mental health unanimously deteriorates across the nation. To address this, we aggregate the individual state-wide results into the mean value of the response variable *K* (for detailed results, see Supplementary Information). If the mean value of $\Delta K = K_{\text {scenario under consideration}} - K_{\text {base case scenario}}$ is $(+)ve$, then the perturbation scenario under consideration is depicted as the worst-case scenario. Similarly, if the mean $\Delta K$ is found to be $(-)ve$, then there is a decline in the percentage of the adults reporting mental health issues, so the scenario is termed as a best-case scenario.

## Discussion

This study employs a library of supervised interpretable machine learning models and scenario-based sensitivity analyses to explore the relationship between adults’ mental health, and the socio-economic and physical aspects of the built environment in the US largest metropolitan areas. The interpretable machine learning models and scenario-based analyses elicit three essential issues for discussion and serve as crucial conversation points for policy discourses and future research.

First, the built environment’s socio-economic aspects are vital to understanding the social determinants of adults’ mental health in metropolitan communities across the US. The interpretable machine learning models suggest that increasing poverty and unemployment levels are associated with a significant increase in adults reporting mental health disorders. The scenario-based analysis supports this finding by showing that declining economic conditions within metropolitan areas are expected to increase the number of adults reporting mental disorders, and this is pronounced in metropolitan areas within states such as Georgia, Massachusetts, Kentucky, Michigan, Ohio, and Wisconsin. A number of studies have long observed the impact of poor economic conditions, manifesting in issues such as poverty, low-income, and unemployment, on mental health^16,49,50,67^. This paper provides evidence to support such existing findings across multiple metropolitan areas, and it allows for both within and across the states comparisons for policy conversations around how to center discussions on community mental health within economic policies at local, state, and national levels.

Second, the results from both the interpretable machine learning models and scenario-based analysis provide an opening to conversations around health insurance and mental health. The debate in the literature focuses on whether or not access to mental health insurance schemes improves the likelihood of a person accessing mental health services, which leads to improved mental health outcomes. While the evidence seems inconclusive based on contradictory studies across countries^51,52,68^, the partial dependence analysis of the health insurance variables in our study show that there is a strong increasing trend between lack of health insurance and adults reporting mental health disorders in metropolitan areas across the states in the US. This analysis goes a step further to show that decreased access to public health insurance is linked to increased mental disorders reported. The scenario-based analysis showed Vermont, the first state to adopt universal healthcare, as an outlier case. Increased access to public health insurance was linked to a significant decrease in mental health disorders reported within Vermont’s largest metropolitan area. This finding does not necessarily suggest the need for universal healthcare. At the very least, it calls for an in-depth research inquiry and policy discourses around how the lack of health insurance, a critical socio-economic need, can impact a person’s mental health.

Finally, the physical aspects of the built environment are found to have mixed impacts on community mental health. Adults report increased mental health disorders as travel costs increase in some metropolitan areas, but this does not hold across all the metropolitan areas in our data sample. Similarly, mental health disorders reported increased as housing vacancy increased in some metropolitan areas, but this also does not hold in all metropolitan areas. The commissioning about the SDGs and mental health rightly observes the need to understand “how neighborhood domain” impact the community mental health. Specifically, it indicates that, besides biological markers, the decline in neighborhood conditions should also be considered as one of the important social determinants of mental health^16^. In conclusion, this article adds to existing studies on how the built environment impacts mental health outcomes^69^, supporting concerns raised in the commissioning report. More importantly, it also adds to the literature on how urbanization (e.g., increasing sprawl and associated commuting costs) impacts mood disorders^70^. The mixed results call for caution when discussing how the built environment’s physical aspects impact community mental health. Future studies may incorporate other physical properties of the built environment such as street density, street connectivity, and land use mix into our proposed multi-level scenario-based predictive analytics framework (MSPAF), to further examine the relationship between the built environment and the community mental health outcomes. Although this article focuses on the large metropolitan areas at a national scale, micro-level data can be collected to explore at a more granular-level, such as intra- and inter-urban and rural dynamics in terms of built environment conditions and mental health outcomes. Future studies may focus more on studying the dynamics within and across urban and rural areas, which remains vital to developing context-specific urban planning, public health and public policy interventions to improve built environment and mental health outcomes.

## Data and methods

### Data collection and pre-processing

In this study, we conducted a nation-level study for all the metropolitan regions in 50 states across the US. We obtained and aggregated data for public health characteristics, built environment features, and socio-economic conditions from multiple sources. From the US Centers for Disease Control and Prevention (CDC) Behavioral Risk Factor Surveillance System (BRFSS), information about the health-related variables like, mental health conditions, pre-clinical conditions and behavioral factors for the adults aged 18 or above are collected at a census tract level for the year 2014^71^. The housing vacancy data for the year 2014 is obtained from US Housing and Urban Development (HUD) at a census tract level^72^. Finally, the socio-economic characteristics like, race, income, unemployment rate, marital status, education level, and access to health insurance information are obtained for the census tract and metropolitan levels from the American Community Survey (ACS) for the years 2011 to 2015^73^. The travel cost data is obtained from the US Department of Housing and Urban Development Low Transportation Cost Index (LAI)^72^, which uses data on housing costs from the American Community Survey (ACS) and estimates transportation costs based on land use mix, commute patterns, and socio-economic information. The data from the multiple sources are matched and aggregated to create the final data set. In our analysis, *the percentage of participants who were adults aged 18 years or more and reported that they were suffering from mental health issues for more than 14 days in the last month* is considered as the response variable. The other variables on health characteristics, built environment features and socio-economic characteristics are considered as the predictors or independent variables. Out of all the categories of the predictor variables, the pre-clinical health condition related variables are found to be highly correlated. To consider the effect of all the pre-clinical variables while having a bound on the number of dimensions, we performed principal component analysis (PCA) (see Supplementary Information). PCA is an unsupervised learning method that uses orthogonal transformations to convert a multidimensional data set of observations of possibly correlated variables into a new multidimensional data set of values of linearly uncorrelated variables^74^. PCA is useful for dimension reduction purpose, because a fewer orthogonal components of the transformed data can capture most of the variance of the original data. In this research, we considered three principal components as they were able to express $92\%$ variability of the observations of the original 12 pre-clinical health related variables taken into consideration.

### Overview of statistical learning

Given a dataset with a response variable *Y* and a set of *p* predictor variables $X = X_1, X_2, \ldots , X_p$, interpretable machine learning algorithms try to identify the function *f* that relates the predictors with the response variable as, $Y = f(X) + \epsilon$^26^. Here, $\epsilon$ is the irreducible error term that arises from unobserved heterogeneity from the data and is normally distributed $N(\mu ,{\sigma }^2)$ where, $\mu$ = mean and ${\sigma }^2$ = variance^25^. Using the *training data* which is a known set of data points, a model is trained to estimate *f* and using an unknown set of data points known as *test data*, the performance of the model is evaluated. In this study, we implemented a suite of interpretable machine learning models, which can be crudely classified into three categories, viz. (i) parametric models, (ii) semi-parametric models and (iii) non-parametric models. In parametric models, the problem of estimating the unknown function *f* gets reduced to estimating a set of parameters through which the model is represented. On the other hand, the non parametric models make no assumption about the unknown function. A semi-parametric model is a hybrid of parametric and non-parametric models. More specifically, we implemented the following algorithms—

1. *Parametric models* Generalized Linear model^38^, Ridge regression^39^ and Lasso regression^40^
2. *Semi-parametric models* Generalized additive model^41^, multi adaptive regression splines^42^,
3. *Non-parametric models* Random forest^44^ and gradient boosting method^43^ Bayesian additive regression trees^45^

To achieve optimal generalization performance for an interpretable machine learning model, it’s complexity should be controlled using the bias-variance trade off technique. Cross validation is the most widely used technique for balancing models’ bias and variance. In this study, the best model was selected using an 80–20 randomized percentage holdout cross validation technique, where the models were trained on randomly selected $80\%$ of the data set and the remaining $20\%$ of the data set were used as holdout set to assess the out-of-sample predictive performance of the models. This technique is repeated 30 times to ensure each data point of the original data set is used at least once for training the models. The metrics used to compare the performances of the models are $R^2$, *RMSE* (root mean squared error) and *MAE* (mean absolute error). This method of model selection is a well-established method and has been used in various previous studies^7,27–30,32–34^. In the following section, we described the Bayesian additive regression trees, which is the best model found in our analysis, and leave the discussion on other methods in the Supplementary Information.

### Bayesian additive regression trees

Bayesian additive regression tree (BART) is a sum-of-trees model where the outputs from *m* ‘small’ decision trees are aggregated with an underlying Bayesian probability model to generate the response function^45,75^. Mathematically, BART can be expressed as,

1 $$\begin{aligned} Y = \left[\sum _{j=1}^m g(X; T_j, M_j)\right] + \epsilon \quad \epsilon \sim N(0,\sigma ^2) \end{aligned}.$$

There are *m* distinct regression trees $T_j$ with their terminal node parameters $M_j$. The function $g(X; T_j, M_j)$ assigns the leaf node parameters *M* of tree *T* to the independent variables *X* for all *m* trees. The main difference of BART compared to other tree ensemble methods is that, BART develops on an underlying Bayesian probability model and consists of a prior, likelihood and posterior probability space. The prior terms are responsible for the tree structure, model complexity, regularization and incorporating expert knowledge in the model. Generally, the Metropolis–Hastings algorithm is used to generate draws from the posterior probability space.

### Model inference

Although the non-parametric models outperform parametric models in terms of predictive performance, the improved predictability comes at the cost of reduced interpretability. However, statistical inferencing can be conducted for the non parametric models using the variable importance ranking and partial dependence plots (PDPs)^26,45,75^. The importance of the variables are depicted by the inclusion proportion of the variables which denote the number of times a particular variable has been selected to develop the model. To understand how a particular predictor variable affects the response variable, the PDPs are used. The PDP is estimated as follows:

2 $$\begin{aligned} p_j(x_j) = \frac{1}{n}\sum _{i=1}^n p_j(x_j, x_{-j},i) \end{aligned}.$$

Here, *p* is the statistical response surface; *n* denotes the number of observations, $x_{-j}$ represents all the independent variables except $x_j$.

### Scenario-based sensitivity analysis

The scenario-based sensitivity analysis implemented in this study involves a systematic approach of statistical simulation. First, the independent variable or the set of independent variables for which the scenario is to be created are selected. For each state, the best parametric distribution that fits the sample data of independent variables (predictors) is identified using the Chi-squared goodness of fit and method of moments for parameter estimation^76^. After the best distribution(s) of the predictors(s) is identified, for each state random sampling is implemented to obtain the base case values (*BV*). Then, according to the hypothesized scenario, the mean of the historical parametric distribution of the variable of interest is perturbed. Then, using random sampling, new values are obtained from the new distribution with the shifted mean, which corresponds to the hypothesized scenario. The original values of the variable are then substituted by the new values corresponding to the scenario while keeping all the other variables same as original. Following this, using the selected statistical learning model, the percentage of population reporting poor mental health are predicted for the new data set. Finally, we identify whether any significant nation-level and/or state-level increase or decrease in the response (compared to the original response variable) is observed or not.

As described before, in this paper, we considered five categories of variables representing socio-economic and physical aspects of a built environment: (i) the economic status of a community characterized by incidence of poverty, unemployment rate and household income, (ii) % of families in a community with no health insurance, (iii) access to public health insurance, (iv) transport cost expressed as a % of income spent towards transportation, and (v) housing vacancy. The mean of each variable’s historical distribution is perturbed $1\sigma$ (standard deviation) of the variable. Corresponding to these sets of variables, ten hypotheses are created (see Table 2).

For each category of the independent variables, we validate our hypotheses by predicting $K_{\text {scenario of hypothesis}}$ denoting the “% adults aged $>18$ suffering from poor mental health for $>14$ days” under the specific scenario of independent variable perturbation (e.g., economic depression) considered for a particular hypothesis (e.g., *H*1). The change in the response corresponding to this perturbed condition is captured by,

$$\begin{aligned} \Delta K = K_{\text {scenario of hypothesis}} - K_{\text {base case scenario}} \end{aligned}.$$

To normalize the effect of the base line response value, we consider $\Delta \kappa$ which captures the projected change in % of adults aged $>18$ years reporting poor mental health for $>14$ days and expressed as a percentage of the baseline estimates.

$$\begin{aligned} \Delta \kappa = \frac{K_{\text {scenario of hypothesis}} - K_{\text {base case scenario}}}{K_{\text {base case scenario}}} \times 100\% \end{aligned}.$$

In Figs. 3, 4, 5, 6 and 7, the output of the sensitivity analysis has been depicted. The $\Delta K$ is plotted in part (b) of each figure, representing the exact projected change in *K*. For each figure, in part (a), the $\Delta \kappa$ is plotted as the bars representing the projected change expressed as a percentage of the baseline estimate with the underlying $K_{\text {base case scenario}}$ depicted in the map as gray scale intensities. In the subsequent sections, we discuss the result of the sensitivity of *K* to different categories of independent variables.

## Supplementary information

Supplementary Information.
