# Supplementary material for: A multilevel scenario based predictive analytics framework to model the community mental health and built environment nexus
Source: Sci Rep. 2021 Sep 2;11:17548. doi: 10.1038/s41598-021-96801-x (PMC8413383; doi:10.1038/s41598-021-96801-x)
Supplement: Supplementary file 1 — Supplementary Information. [file 41598_2021_96801_MOESM1_ESM.pdf]

Supplementary Information for “A multilevel scenario based predictive analytics framework to model the community mental health and built environment nexus"

Sayanti Mukherjee[1,*], Emmanuel Frimpong Boamah[2], Prasangsha Ganguly[1], and Nisha Botchwey[3]

[1]University at Buffalo - The State University of New York, School of Engineering and Applied Sciences, Department of Industrial and Systems Engineering, Buffalo NY, 14260, U.S.A.
[2]University at Buffalo - The State University of New York, School of Architecture and Planning, Department of Urban and Regional Planning, Buffalo NY, 14214, U.S.A.
[3]Georgia Institute of Technology, School of City & Regional Planning, Atlanta GA, 30332, U.S.A.
[*]sayantim@buffalo.edu

ABSTRACT

Contents of this file include:

Methods: Dimensionality reduction of pre-clinical health conditions: Principal component analysis
Methods: Methodological background on various statistical learning models
Figures 1-2: Principal Component Analysis Results
Figure 3: Variable Importance Plot
Table 1: Sensitivity Analysis Results

Dimensionality reduction of pre-clinical health conditions: Principal component analysis

In our analysis, we considered twelve pre-clinical health conditions. They are, cancer, high cholesterol level, coronary heart disease, arthritis, high blood pressure, stroke, chronic kidney disease, chronic obstructive pulmonary disease (COPD), diabetes, obesity, asthma, and the mental health issues for up to fourteen days. To reduce the dimensions, we performed the principal component analysis (PCA). PCA is an orthogonal transformation of the data into a new coordinate system that explains the maximum variances in the data. The results obtained from the principal component analysis for dimension reduction of the pre-existing health conditions are described. The Fig. 1, depicts the correlation circle plot of the pre-clinical health conditions. The contribution of the variables (contrib) are depicted along with the variance explained by the dimensions.

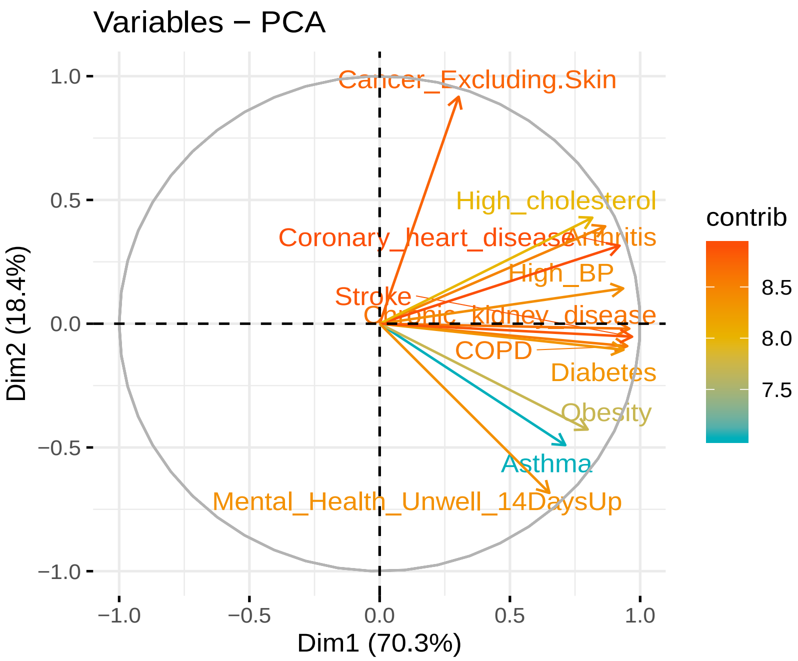

Figure 1. The correlation circle plot of the pre-clinical conditions

Furthermore, to identify the number of reduced dimensions, we plotted the scree plot in Fig. 2. In multivariate statistics, scree plot depicts the line plot of the eigenvalues or the percentage of explained variance of the principal components. From the plot, it can be identified that, by choosing three dimensions, most of the variance has been explained. The top three dimensions can explain 92% of the variance and hence this is considered as the output.

Methodological background on various statistical learning models

In this section, the methodological backgrounds of different statistical learning models other than the Bayesian Additive Regression Trees (BART) used in our analysis are described. More specifically, here we discuss the methodological backgrounds of the generalized linear model (GLM), ridge regression, lasso regression, generalized additive model (GAM), multivariate additive regression splines (MARS), random forest (RF) and gradient boosting method (GBM).

Generalized linear model (GLM)

A GLM is an extension of the linear regression where the normality assumption of the error terms is relaxed. There are three components of a GLM: an exponential family of probability distributions, a systematic component and a link function. In this setting, the dependent variable Y belongs to an exponential family which can be expressed as,

$$Y \sim f_Y(y_i)$$

$$f_Y(y_i) = exp\{\frac{y_i\theta_i - b(\theta_i)}{a(\phi)} + c(y_i,\phi)\}$$

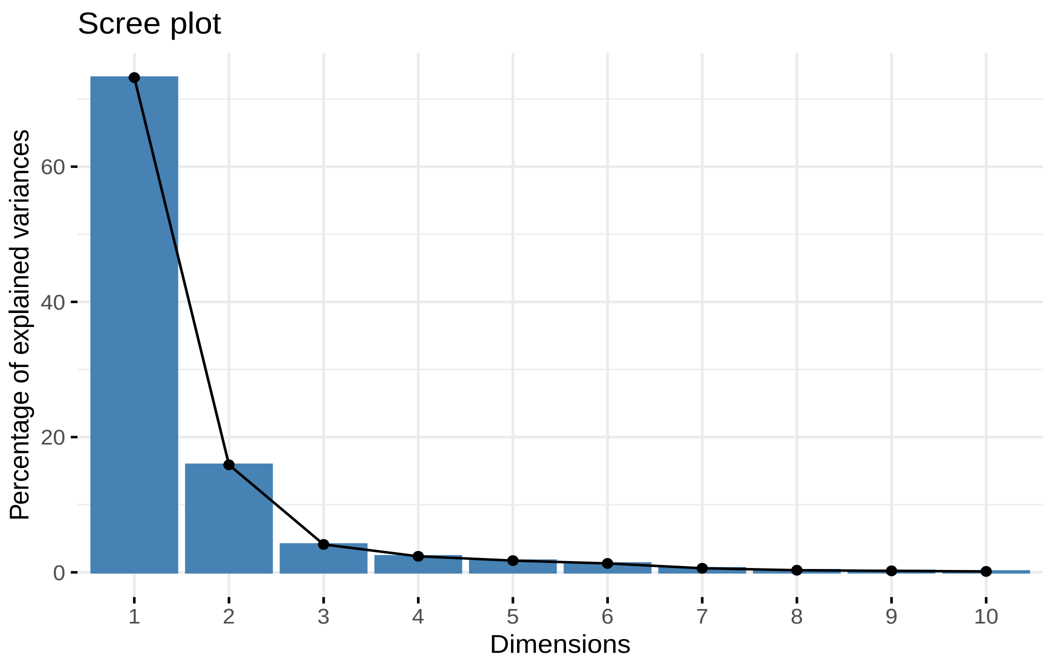

Figure 2. The scree plot of the pre-clinical conditions

Where, $x_{i1}, x_{i2}, \ldots, x_{ip}$ is the set of p dimensional predictors and y_i is the corresponding response value; $f_Y(y_i)$ is the probability density function of Y, θ_i is a function of $x_{i1}, x_{i2}, \ldots, x_{ip}$ known as the natural parameter; and ϕ is called the scale parameter which is constant for all the observations i.

The systematic component is a linear combination of the predictor variables can be expressed as,

$$\eta = \beta_0 + \sum_{j=1}^{p} \beta_j x_{ij}$$

This systematic component η is linked to the response through a link function $g()$ such that, $\mathbf{E}(Y|X) = g^{-1}(\eta)$.

Essentially, the GLM is highly interpretable and easy to fit. However, due to its parametric nature, the predictive accuracy of these models is less compared to other semi-parametric or non-parametric methods.

Ridge regression

In least square estimates, the variance of the model increases with increase in number of predictors, and some of the predictor variables may not have a significant effect on the response. Ridge regression is a parametric method where, the coefficients are estimated by minimizing the sum of the residual sum of squares and a shrinkage penalty. The estimated coefficients of ridge regression are calculated by minimizing

$$\sum_{i=1}^{n} (y_i - \beta_0 - \sum_{j=1}^{p} \beta_j x_{ij})^2 + \lambda \sum_{j=1}^{p} \beta_j^2 \tag{1}$$

Essentially, the shrinkage penalty $(\lambda \sum_{j=1}^{p} \beta_j^2)$ has an effect of reducing the estimates of β_j towards 0. The tuning parameter λ controls the shrinking parameters and it is estimated using cross-validation.

In a ridge regression setting, the coefficients are reduced but never made equal to 0 unless λ is infinitely large. Hence, the ridge regression will always have all the p predictors in it.

Lasso regression

The Lasso regression is an improvement over the ridge regression that is capable of reducing the coefficients of some predictors to 0. The lasso coefficients are estimated by minimizing,

$$\sum_{i=1}^{n} (y_i - \beta_0 - \sum_{j=1}^{p} \beta_j x_{ij})^2 + \lambda \sum_{j=1}^{p} |\beta_j| \tag{2}$$

When the tuning parameter λ is sufficiently large, some of the coefficients are reduced to 0. Lasso regression can perform variable selection and the models generated by this model are more interpretable.

Generalized Additive Model (GAM)

The GAM is a semi-parametric method that allows non-linear functions for each variable to be added to generate a final model. It is essentially an extension of multiple regression, where to allow non-linear relationships between each of the p predictors (x_{ij}) and the response (y_i), a smooth non-linear function ($f_j(x_{ij})$ is introduced. In this setting, for each variable, a non-linear function (f_j) is estimated non-parametrically and then added to generate the final model. The model can be expressed as,

$$y_i = \beta_0 + \sum_{j=1}^{p} f_j(x_{ij}) + \varepsilon_i$$

Being a more flexible model compared to the parametric models, GAM is able to make superior predictions. However, it is prone to overfit the data if proper cross-validation is not performed.

Multivariate Adaptive Regression Splines (MARS)

MARS is a semi-parametric regression technique that can model non-linearities and particularly suitable for high dimensional data sets. It can be represented as a sum of splines where the response variable is allowed to vary non-linearly with the predictor variables. The model can be represented as,

$$f(X) = \beta_0 + \sum_{m=1}^{M} \beta_m h_m(X)$$

where the output is $f(X)$; β_0 is the intercept; the spline for each predictor is $h_m(x)$ and β_m is the vector of coefficients estimated by minimizing the sum of square errors.

Random forest (RF)

Random Forest is a non-parametric ensemble tree based method. The method ensembles B bootstrapped regression trees (T_b) where B is selected based on cross-validation. The final estimate is made by averaging the predictions across all trees as shown in the equation below.

$$f^B(X) = \frac{1}{B} \sum_{b=1}^{B} T_b(X)$$

The random forest are low bias techniques, i.e they can capture the pattern of the data very well. However, these models have high variance and sensitive to outliers.

Variable importance plot from BART

The variable importance plot depict the inclusion proportion of the variables in Fig 3.

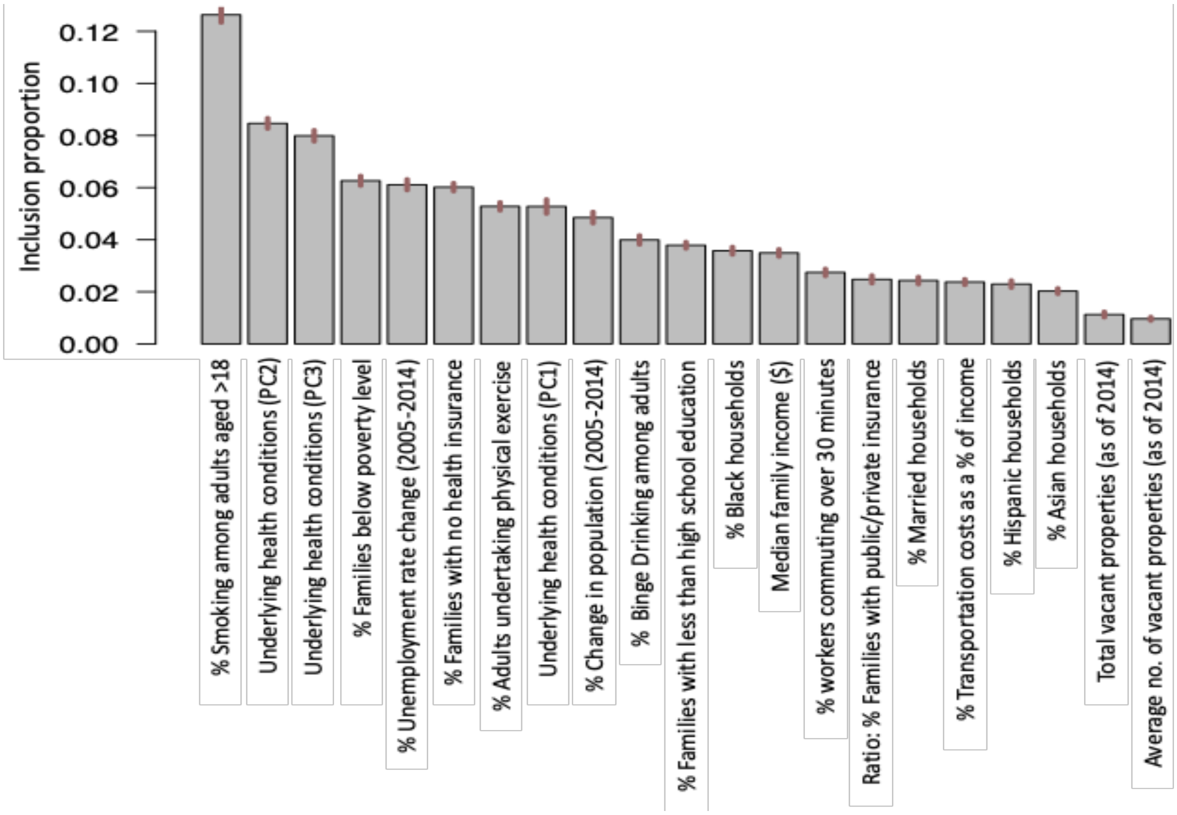

Figure 3. Ranking of variable importance.

Sensitivity analysis results

Perturbation scenario	Mean $K_{\text{perturbation scenario}}$	Mean $K_{\text{base case scenario}}$	Mean ΔK	Conclusion
Economic degradation	13.75	13.27	0.48	**Worst case scenario**
Economic improvement	13.0	13.27	−0.27	**Best case scenario**
Less unavailability of health insurance:	12.78	13.31	−0.53	**Best case scenario**
More unavailability of health insurance:	13.84	13.31	0.53	**Worst case scenario**
Decreased access to public health insurance	13.37	13.29	0.08	**Worst case scenario**
Increased access to public health insurance	13.22	13.29	−0.07	**Best case scenario**
Cheaper mode of travel and/or shorter commuting distance to work:	13.27	13.29	−0.02	**Best case scenario**
Expensive mode of travel and/or longer commuting distance to work:	13.30	13.29	0.01	**Worst case scenario**
Community expanding or, vacancy decreasing	13.31	13.30	0.01	**Worst case scenario**
Community shrinking or, vacancy increasing	13.29	13.30	−0.01	**Best case scenario**

Table 1. Aggregated result
